# Fast Clearance of the SARS-CoV-2 Virus in a Patient Undergoing Vaccine Immunotherapy for Metastatic Chordoma: A Case Report

**DOI:** 10.3389/fonc.2020.603248

**Published:** 2020-11-16

**Authors:** Danielle M. Pastor, Katherine Lee-Wisdom, Andrew E. Arai, Arlene Sirajuddin, Douglas R. Rosing, Borys Korchin, James L. Gulley, Marijo Bilusic

**Affiliations:** ^1^Medical Oncology Service, Center for Cancer Research, National Cancer Institute, National Institutes of Health, Bethesda, MD, United States; ^2^Cardiovascular Branch, National Heart, Lung, and Blood Institute, National Institutes of Health, Bethesda, MD, United States; ^3^Bavarian Nordic, Morrisville, NC, United States; ^4^Genitourinary Malignancies Branch, Center for Cancer Research, National Cancer Institute, National Institutes of Health, Bethesda, MD, United States

**Keywords:** severe acute respiratory syndrome coronavirus 2, coronavirus disease 2019, chordoma, modified vaccinia Ankara-brachyury vaccine, immunotherapy

## Abstract

The emergence of the SARS-CoV-2 virus has been associated with perplexing clinical sequelae and phenomena that often have no clear link to the underlying infection. There is a wide spectrum of symptoms associated with infection, from minimal respiratory complaints to severe multi-organ failure, often resulting in death. Individuals with malignancies, particularly those whose treatments have left them immunocompromised or immunosuppressed, are among the patient populations thought to be at greater risk for more severe illness. A man with aggressive metastatic chordoma contracted the SARS-CoV-2 virus and was diagnosed with COVID-19 while undergoing intravenous brachyury vaccine immunotherapy. His disease course was remarkably mild, and the virus cleared rapidly. Despite a treatment delay of 3 months due to the COVID-19 pandemic, the patient’s disease has been stable and tumor-related pain has significantly improved. This suggests not only an intact, functional immune system, but also one that appears to have been responsive to cancer treatment. It has been suggested that individuals undergoing treatment for metastatic cancer are at greater risk of severe SARS-CoV-2-related illnesses and complications. While immunosuppression may be a problem, particularly in those receiving conventional chemotherapeutic agents, it is possible that the non-specific effects of immune-enhancing therapies may confer some protection against SARS-CoV-2.

## Introduction

Reports from the biomedical community have suggested that cancer patients are at increased risk for severe illness from coronavirus disease 2019 (COVID-19) and more vulnerable to associated complications (1, 2). We present the case of a patient with metastatic chordoma who contracted the severe acute respiratory syndrome coronavirus 2 (SARS-CoV-2) virus and developed COVID-19 disease during treatment with vaccine immunotherapy. His unexpectedly mild and short-lived course of disease may conceivably have been, in part, attributed to the immune-modulating effects of his vaccine therapy. This case report calls attention to the concept of potential protection from acute infection conferred by the effects of therapeutic vaccination in the treatment of malignancy.

## Case Report

**Figure 1:**
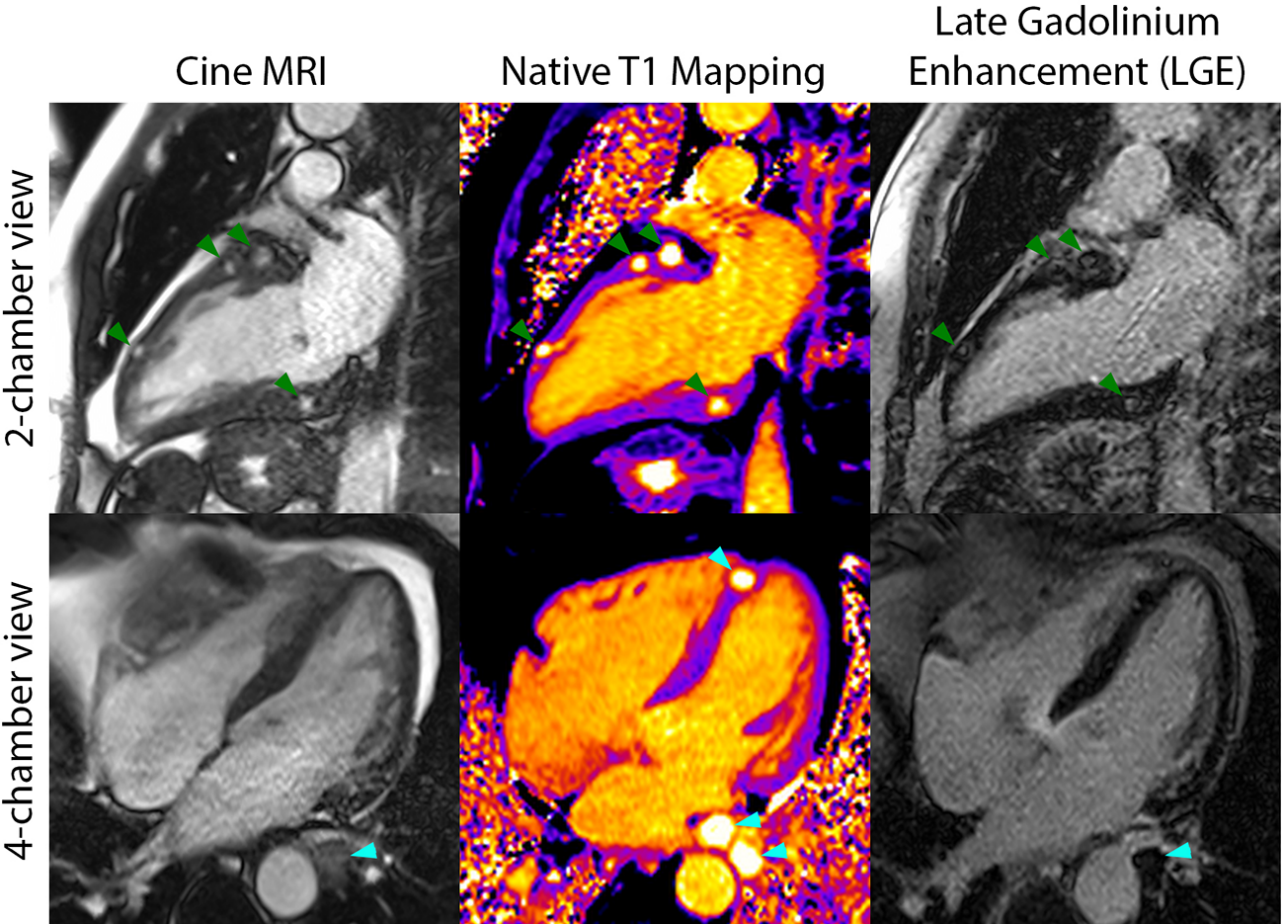
Cardiac magnetic resonance imaging included cine MRI (left column), native T1 mapping (middle column), and late gadolinium enhancement (LGE) imaging (right column) and demonstrated several myocardial metastases on the two-chamber views (green arrows) and three metastases in the four-chamber views (cyan arrows). The metastases were best seen on T1 mapping and cine MRI and had long T1 and T2 (not shown) relative to myocardium.

A 60-year-old man was diagnosed with widely metastatic sacral chordoma on 01/02/2019 and treated with volumetric modulated arc radiotherapy and proton beam radiation from 02/21/2019 to 04/10/2019. His disease progressed shortly thereafter, and he was started on pembrolizumab monotherapy on 07/17/2019. He developed enlarging bilateral pulmonary metastases and new hepatic lesions on 01/10/2020. At an evaluation at the National Cancer Institute on 02/25/2020, imaging revealed widely metastatic disease with multiple new hypodense left ventricular myocardial lesions. Echocardiogram and cardiac magnetic resonance imaging confirmed the presence of at least 7 distinct myocardial lesions of the left atrium and left ventricle, ranging from 0.65 cm x 0.65 cm to 2.0 cm x 1.6 cm, with preservation of the left ventricular ejection fraction (**Figure 1**). History and physical exam revealed an independently functioning individual with hypertension as his only pre-existing comorbidity who possessed good activity tolerance, limited only by bladder and bowel incontinence and neuropathy directly related to sacral tumor compression. His medications included gabapentin, immediate-release morphine sulfate, and methadone. Lab work was notable for a hemoglobin level of 11.8 g/dl and a lactate dehydrogenase level of 281 U/L. He was deemed eligible for a first in human clinical trial and received his first dose of modified vaccinia Ankara-Bavarian Nordic (MVA-BN)^®^-based brachyury intravenous vaccine (NCT04134312) on 03/11/2020. The patient’s post-treatment course was remarkable for transient grade 1 fever and chills attributed to his intravenous vaccine administration and left upper extremity blisters localized to the distal forearm, attributed at that time to a contact dermatitis, which resolved with topical fluocinonide ointment.

The patient arrived 3 weeks later for his planned second vaccination presenting with new anorexia associated with a 12-pound weight loss, worsening fatigue, and a new, non-productive cough, all of which had started 12 days prior to his presentation. In that time, he had had no gastrointestinal symptoms, dyspnea, or fevers. Physical exam was notable only for the patient’s thin stature and fatigued appearance; breath sounds were normal. Laboratory findings revealed high-sensitivity c-reactive protein 138 mg/L, ferritin 1,627 mcg/L, hemoglobin 11.1 g/dl, platelet count 410 K/mcl, absolute neutrophil count 6.56 K/mcl, and absolute lymphocyte count 0.49 K/mcl (**Table 1**). Chest x-ray demonstrated mild bilateral pulmonary opacities and bibasilar atelectatic changes. COVID-19 was diagnosed based on the detection of SARS-CoV-2 RNA from a nasopharyngeal sample by reverse transcriptase polymerase chain reaction (RT-PCR) performed using the Centers for Disease Control and Prevention (CDC) 2019-novel coronavirus (2019-nCoV) Real-Time RT-PCR Diagnostic Panel (3). His second dose of treatment vaccine was held, and he was admitted for supportive treatment with intravenous fluid and antitussive agents. Several days into his hospital course, a chest CT scan revealed the interval development of bilateral ground glass lung infiltrates in the right upper lobe, lingula, and bilateral lower lobes, as well as peribronchial infiltrates associated with bronchial wall and septal thickening. On the patient’s fifth hospital day, RT-PCR testing was negative for the presence of viral RNA. It remained undetected each of the next 2 days, and the patient was discharged in good condition on hospital day 7. He remained hemodynamically stable and maintained normal oxygen saturation on room air throughout his entire hospital stay.

**Table 1:** Relevant laboratory data.

	Baseline	Post-first dose treatment	COVID-19admission	Discharge
**SARS-CoV-2 RNA**	—–	—–	**Positive**	**Negative**
**High sensitivity C-reactive protein (mg/L)**	18.0	17.8	138.4	16.6
**Ferritin (mcg/L)**	—–	—–	1,627	758
**D-dimer (mcg/mL FEU)**	—–	—–	—–	4.23
**Thyroid stimulating hormone (mIU/ml)**	2.70	1.10	1.50	—–
**Glucose (mg/dl)**	95	91	123	89
**Lactate dehydrogenase (U/L)**	281	213	304	—–
**Uric acid (mg/dl)**	6.3	6.0	7.0	—–
**White blood cell count (K/mcl)**	5.92	3.17	7.66	4.56
**Hemoglobin (g/dl)**	11.8	10.7	11.1	9.9
**Platelets (K/mcl)**	281	235	410	303
**Absolute neutrophil count (cells/mcl)**	2,860	1,320	6,560	2,590
**Absolute lymphocyte count (cells/mcl)**	1,320	710	490	1,450
**Neutrophil to lymphocyte ratio**	2.17	1.86	13.38	1.79
**Lymphocyte to C-reactive protein ratio**	733.33	398.88	35.51	873.49

**Figure 2:**
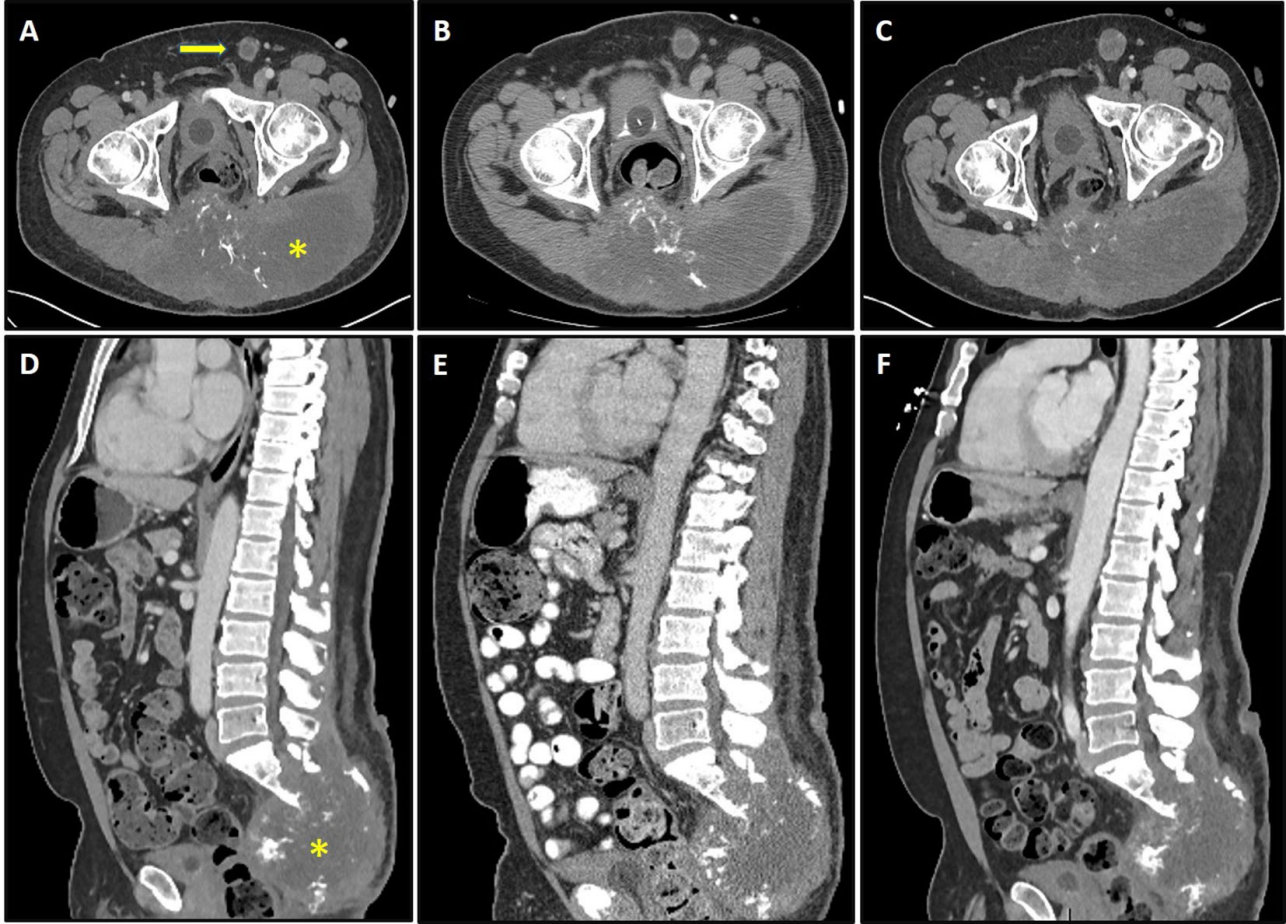
Axial and sagittal sections of CT scans depicting sacral chordoma (asterisk) with left inguinal lymphadenopathy (arrow) prior to brachyury vaccine **(A, D)**, 4 weeks after the first dose of vaccine **(B, E)**, and 16 weeks after the first dose of vaccine **(C, F)**. Imaging demonstrates stable disease by response evaluation criteria in solid tumors (RECIST) criteria, with 1.54 and 3.38% changes in baseline at 4 weeks **(B, E)** and 16 weeks **(C, F)**, respectively.

On a follow-up phone call after his discharge, the patient reported feeling substantially better, with no more fatigue and a normal appetite. Remarkably, the patient’s tumor-associated pain had also significantly improved, such that he had discontinued his methadone and was able to significantly decrease his dose of immediate-release morphine. Restaging CT scans at that time demonstrated stable disease (**Figure 2**). Although he felt quite well overall, his rash recurred on 05/05/2020, this time more extensive with large, tense bullae involving all four extremities. Punch biopsies confirmed bullous pemphigoid. Sera collected at baseline (prior to vaccine treatment) was evaluated for autoantibodies to BP180 and was found to be positive, with an elevated level of 173 RU/ml, consistent with immune checkpoint inhibitor-induced bullous pemphigoid secondary to prior pembrolizumab treatment. The patient responded well to oral steroids and was restarted on vaccine treatment on 07/01/2020.

## Discussion

Chordoma is a rare tumor of notochordal origin with potential to involve any portion of the entire central neuroaxis. While generally slow-growing, chordoma can be locally aggressive and result in significant comorbidity. Surgery and radiotherapy are mainstays of treatment, while chemotherapy is ineffective. Our patient’s case is unique in that he not only had rapidly progressive chordoma, but he had also developed multifocal myocardial metastases. Cardiac metastases from chordoma are very rare; multifocal disease in multiple chambers is even more rare, with only a few case reports in the literature (4). Multiple studies have demonstrated the expression of programmed cell death ligand-1 (PD-L1) in chordoma; moreover, some have shown success with the use of immune checkpoint blockade in the treatment of chordoma with varying levels of clinical response and durability (5–8). Disease response has been observed in patients treated with a yeast-based brachyury vaccine (9). Brachyury, thought to be overexpressed in >95% of chordomas, as well as in a spectrum of other human carcinomas, is considered a driver of mesenchymalization, metastatic potential, and therapeutic resistance (10). Patients with advanced solid tumors of various types, including chordoma, who were treated with the yeast-brachyury vaccine were shown to mount brachyury-specific T-cell immune responses, with activation of both CD4^+^ and CD8^+^ cells and increased intracellular expression of IFN gamma, TNF alpha, and IL2, as well as CD107a (9, 11). Further, T cell responses appeared to increase with escalating vaccine dose (9). In consideration of brachyury expression in chordoma and its role in pathogenesis, the BN-brachyury vaccine was granted orphan drug designation for the treatment of chordoma by the U.S. Food and Drug Administration in May 2018 (12).

Our patient was diagnosed with COVID-19 during treatment with BN-brachyury, a poxvirus-based vaccine. Interestingly, his presentation and course were different than those described in similar patients with cancer who subsequently developed COVID-19. While much remains unknown about the various manifestations of COVID-19, several factors have been identified that appear to correlate with higher risk of disease severity and complications. Multiple studies have reported that patients with malignancy have a higher risk of severe COVID infection-related events, including hospitalization, invasive ventilation, and death (1, 2). Given what we know and continue to learn about COVID-19 risk factors, our patient would be expected to have a more severe or more prolonged disease course. However, his case was remarkably uncomplicated, prompting us to ask whether the non-specific immunostimulatory effects generated by the tumor-antigen vaccine he received might have contributed to his milder disease phenotype and faster viral clearance.

The susceptibility of cancer patients to higher risks of SARS-CoV-2 infections and COVID-19-related complications may be the result of several factors, including the acquired systemic immunodeficiency arising in the setting of toxic anti-cancer treatment, as well as an immunosuppressive microenvironment supporting tumor immune evasion or escape (13). The use of conventional chemotherapy may certainly result in immunosuppression; however, in recent years, additional anti-cancer treatments have emerged that differ in their toxicity profiles and effects on the immune system. Immune checkpoint inhibitors and therapeutic cancer vaccines are immunomodulating agents with capacity to restore cell-mediated immunocompetence. Recently, a prospective study assessing the effect of various anti-cancer treatments on mortality in cancer patients with COVID-19 found no significant effect on mortality for use of immune checkpoint inhibition, targeted therapy, radiotherapy, or hormone therapy within 4 weeks of COVID-19 diagnosis (14). Further, as discussed in a review by Vivarelli *et al*., there has been no direct evidence of immune checkpoint inhibitor-induced toxicity and increased viral infection risk in cancer patients, prior or during the pandemic (15). These findings not only support the consideration of continued use of immune checkpoint inhibitors in the treatment of malignancy on a case-by-case approach throughout the current pandemic, but also support the study of the potential benefit of the use of these agents in reestablishing cell-mediated immunocompetence in the setting of SARS-CoV infection.

The MVA-BN-brachyury vaccine is a novel recombinant therapeutic cancer vaccine composed of a replication-deficient attenuated derivative of the vaccinia virus as its vector (16). Designed to induce an enhanced immune response against the transcription factor brachyury, the vaccine encodes the human brachyury gene in combination with a triad of human T-cell costimulatory molecules (TRICOM): human lymphocyte function-associated antigen 3 (hLFA-3 or CD58), the human intercellular adhesion molecule 1 (hICAM-1 or CD54) and hB7.1 (or CD80). Investigators have demonstrated the ability of this vector-based vaccine to activate CD8^+^ and CD4^+^ T cells specific against the antigen brachyury, both *in vitro* and in patients with advanced cancer, when administered subcutaneously (16). *In vitro*, activation of brachyury-specific T cells by MVA-brachyury-TRICOM–infected dendritic cells was confirmed by proliferation and IFN gamma production in response to brachyury protein expression. Subsequent Phase I data demonstrated that 82% of patients vaccinated with MVA-brachyury-TRICOM developed brachyury-specific T cell responses post-vaccination with no dose-limiting toxicity, demonstrating the immunogenicity of this antigen (16).

The MVA-BN-Brachyury vaccine has been designed to destroy tumor cells *via* the induction of a highly efficient tumor specific T cell response against tumor-associated antigens. Intravenous delivery of this construct was proposed to allow for the additional activation of the innate immune system, with increased activation of NK cells and cytokine production. Multiple preclinical studies have suggested that the intravenous administration of some vaccines evoke superior NK and T cell responses, as well as higher levels of systemic cytokines, than other routes (17, 18).

Non-specific or off-target effects of vaccines have recently garnered global attention for their potentially protective role in the ongoing COVID-19 pandemic. The heterologous effects of bacillus Calmette-Guerin (BCG) vaccination have been proposed as an underlying explanation for the striking disparity in mortality and morbidity rates among countries that have been severely affected by COVID-19. Epidemiologic studies suggest that a possible critical factor driving this difference is the pre-existing implementation of universal BCG vaccination policies (19, 20). Countries with no national BCG immunization policies in place, such as Italy and Spain, have experienced higher mortality associated with COVID-19, while mortality has been statistically lower in countries with longstanding national BCG vaccination policies, such as China (19, 20). The potential epidemiological correlation between compulsory vaccination policies and reduced COVID-19-associated mortality has fueled intense interest in the possible mitigation of COVID-19-associated processes by the protective heterologous effects of BCG immunization. In fact, multiple clinical trials have already been launched with the intent of examining this link.

It is conceivable that the cross-protection afforded by the antigen-independent activation of bystander B and T cells (heterologous immunity), in conjunction with the immunologic memory developed by innate immunity (trained immunity) might, in part, have contributed to the rapid clearance of the SARS-CoV-2 virus in our patient who underwent treatment with a recombinant MVA-BN-based tumor-antigen vaccine. There is no direct link presently known between the MVA-BN-brachyury vaccine and either the absence of severe symptoms in patients with COVID-19 nor mitigation of disease course. The collective observations noted during our care of a cancer patient with concomitant COVID-19 infection are hypothesis-generating. Further investigation of the interactions between immune system constituents and virus in this setting is necessary to better understand their potential interplay and to develop feasible treatment strategies against this viral illness.

## Data Availability Statement

The original contributions presented in the study are included in the article/supplementary material. Further inquiries can be directed to the corresponding author.

## Ethics Statement

Ethical review and approval was not required for the study on human participants in accordance with the local legislation and institutional requirements. The patients/participants provided their written informed consent to participate in this study. Written informed consent was obtained from the individual(s) for the publication of any potentially identifiable images or data included in this article.

## Author Contributions

DP wrote and edited manuscript, created figures. KL-W assisted in case interpretation and editing of manuscript. AA created cardiac images and assisted with cardiac-related discussion within the manuscript. AS assisted with generation of cardiac images and related discussion. DR provided assistance with interpretation of data and writing of the discussion. BK assisted with discussion related to vaccine therapy. JG was responsible for conception of work, contributed to critical analysis and revision. MB was responsible for conception of work, critical analysis and revision, direction and supervision all above activities, final approval of manuscript for publication. All authors contributed to the article and approved the submitted version.

## Conflict of Interest

Author BK was employed by the company Bavarian Nordic. Author AA had Cooperative Research and Development Agreements with Siemens and Circle CVI and patents pending on quantification of myocardial perfusion by cardiac MRI.

The remaining authors declare that the research was conducted in the absence of any commercial or financial relationships that could be construed as a potential conflict of interest.
